# *edn1* and *hand2* Interact in Early Regulation of Pharyngeal Arch Outgrowth during Zebrafish Development

**DOI:** 10.1371/journal.pone.0067522

**Published:** 2013-06-24

**Authors:** Mark M. Sasaki, James T. Nichols, Charles B. Kimmel

**Affiliations:** Institute of Neuroscience, University of Oregon, Eugene, Oregon, United States of America; Deakin School of Medicine, Australia

## Abstract

Endothelin-1 (Edn1) signaling provides a critical input to development of the embryonic pharygneal arches and their skeletal derivatives, particularly the articulating joints and the ventral skeleton including the lower jaw. Previous work in zebrafish has mostly focused on the role of Edn1 in dorsal-ventral (DV) patterning, but Edn1 signaling must also regulate tissue size, for with severe loss of the pathway the ventral skeleton is not only mispatterned, but is also prominently hypoplastic – reduced in size. Here we use mutational analyses to show that in the early pharyngeal arches, ventral-specific *edn1*-mediated proliferation of neural crest derived cells is required for DV expansion and outgrowth, and that this positive regulation is counterbalanced by a negative one exerted through a pivotal, ventrally expressed Edn1-target gene, *hand2*. We also describe a new morphogenetic cell movement in the ventral first arch, sweeping cells anterior in the arch to the region where the lower jaw forms. This movement is negatively regulated by *hand2* in an apparently *edn1*-independent fashion. These findings point to complexity of regulation by *edn1* and *hand2* at the earliest stages of pharyngeal arch development, in which control of growth and morphogenesis can be genetically separated.

## Introduction

Beginning with mutations isolated in the early large-scale genetic screens in zebrafish [1], [2] a large amount of information has accumulated on the genetic regulation of development of the pharyngeal arches of this species, and the region of the larval skeleton derived from the embryonic arches. Most of the studies focus on patterning of the first two arches, mandibular and hyoid, which by four days postfertilization (dpf), a day after hatching, have both developed cartilaginous elements with articulating joints between them. These elements in the first arch comprise the skeleton of the upper and lower larval jaws, and in the second the supports for the jaws, the buccal cavity, and the operculum that covers the gill chamber. The characterization of zebrafish mutants exhibiting prominent ventral cartilage and joint region defects in the anterior arches [2] complemented earlier work in mouse in identifying the Endothelin1 (Edn1) signaling pathway as being critical for arch patterning [3]. Edn1 secreted by ventral epithelial and arch mesoderm may function in the manner of a morphogen [4], part of the mechanism specifying dorsal-ventral (DV) identity in the neural crest-derived skeletal ectomesenchyme. Edn1-dependent specification includes dynamic and position-dependent upregulation in the responding cells of target genes encoding transcription factors. These include *hand2*, with expression restricted to the ventral patterning domain that forms the ventral cartilage elements, and a set of *Dlx* genes restricted more dorsally to the intermediate domain from which the joints and joint-related skeleton develops [5], [6], [7], [8], [9], [10], [11]. Proper patterning requires cross-talk among the Edn1-target genes. For example *hand2* negatively regulates *Dlx* genes, serving as part of the regulation restricting the *Dlx* genes from more ventral expression [10], [12], [13].

Other recent work in zebrafish has identified additional signaling pathways, Notch and BMP, that interact with Edn1, and these studies fill in gaps in our understanding of arch patterning coming from just Edn1 signaling in isolation [14], [15], [16]. However, apart from arch patterning, the Edn1 signaling pathway must also play a critical role in the regulation of arch and pharyngeal skeleton size, because with severe loss of function of the pathway, particularly the ventral elements of the skeleton can be dramatically reduced. Indeed, the early studies of Edn1 signaling mutants in mouse showed loss of function of *Edn1*, *Ednra*, or *Ece*-*1*, which respectively encode the ligand, receptor, and a ligand processing enzyme, all result in a severely reduced mandible [17], [18], [19], [20]. These reports discuss the function of the pathway as being important for growth or survival of cells in the ventral arches rather than emphasizing regulation of DV patterning. In chick, inhibition of the Ednra receptor through pharmacological methods results in severe reduction of the lower beak and absence of the arch 2-derived hyoid bone [21]. Moreover, loss of a specific Edn1 downstream target gene in mouse, *Hand2* results in hypoplastic first and second arches [5], [22]. Zebrafish *edn1* and *hand2* mutants also have severe reduction of the arch 1 and 2 ventral skeleton [6], [12]. Furthermore, Walker et al. [23] described a substantial reduction in DV elongation of the ventral region of the embryonic pharyngeal arches in zebrafish *edn1* and in *furina* mutants in which maturation of the Edn1 signaling peptide is disrupted. However, it is unknown whether the phenotype is due to change in growth versus morphogenesis.

In this study we use *edn1* and *hand2* mutants to focus on pharyngeal arch size regulation and examine *edn1;hand2* double mutants to learn the epistatic relationships between these two genes. We extend the previous results showing *edn1*-dependent DV expansion, and we find that the expansion is due to growth, i.e., arch increase in size, rather than morphogenetic shape change. Previous work in zebrafish had demonstrated that *edn1* functions upstream to *hand2* in a positive pathway regulating DV patterning [6], hence we were surprised to find that in the early pharyngeal arches, *hand2* functions as a negative regulator of *edn1*-dependent ventral outgrowth. We provide direct evidence that the control of this outgrowth is at least in part by regulation of cell proliferation. Finally, we show that *hand2* negatively regulates an anterior morphogenetic cell movement in the ventral first arch. This regulation appears to be independent of *edn1* function.

## Results

### Epistasis Analysis of Larval Cartilage Phenotypes: an *edn1–hand2* Genetic Pathway

**Figure 1:**
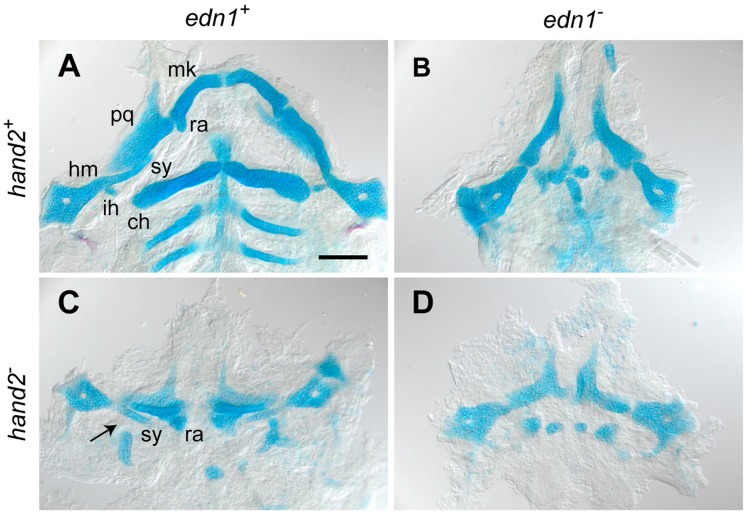
*edn1* and *hand2* are required for ventral jaw cartilage development. 4 dpf zebrafish skeletons were cartilage and bone stained with Alcian Blue and Alizarin Red, dissected and flat-mounted. (A) WT, with cartilages of the first two arches indicated: In the first arch the dorsal palatoquadrate (pq) and ventral Meckel’s cartilage (mk) articulate, Meckel’s cartilage includes a distinctive retroarticular process (ra) adjacent to the joint. In the second arch the more dorsal hyosymplectic cartilage is subdivided into the hyomandibular (hm) and symplectic (sy) regions, and the prominent ventral cartilage is the ceratohyal (ch). The interhyal cartilage (ih) forms a small hinge within the joint region. (B) In the *edn1^−^* larva, ventral cartilages and elements of the joint regions are missing or prominently disrupted. Unidentifiable elements are scattered near the ventral midline. (C) The *hand2^−^* larva exhibits ventral reductions similar to *edn1^−^*, but the arch 2 symplectic cartilage and joint (arrow) are present and the arch 1 retroarticular process is expanded rather than missing. (D) The *edn1^−^;hand2^−^* larva exhibits defects similar to *edn1^−^*; in particular, the symplectic cartilage and retroarticular process cannot be identified. Anterior is upward and right is towards the left. Scale bar: 100 µm.

Previous work has established that with severe loss of function of either *edn1* or *hand2* in zebrafish, developmental patterning of the embryonic pharyngeal arches is disrupted along the DV axis [6], [10], [12], [24]. Subsequently, the arch-derived early larval skeleton is severely malformed (Figure 1). We focus on the first two pharyngeal arches (arch 1, mandibular; arch 2, hyoid). Heterozygotes, including compound heterozygotes have no discernable mutant phenotypes (not shown). The skeletal phenotypes of homozygous *edn1* and *hand2* single mutants are essentially as previously described [6], [10], [12], [24], fully penetrant and broadly similar to each other. However, the phenotypes differ in detail. In the second arch in both single mutants, the hyomandibular dorsal region of the hyosymplectic cartilage is almost normal. In contrast, ventrally both single mutants have variable numbers of small cartilage nodules in the place of ventral ceratohyal cartilage, prominent in the wild type (WT), and the ventral midline basihyal cartilage. Intermediate between the dorsal and ventral elements, the WT forms the symplectic and interhyal cartilages and the articulation (DV joint) between the interhyal and ceratohyal. None of these features are present in the *edn1* single mutant. In the *hand2* single mutant, a recognizable interhyal is absent, but the symplectic and joint are present. Thus it is in this intermediate region where the phenotypes of the two mutants differ.

As we interpret the first arch phenotypes of both single mutants, we also reach the conclusion that the mutants differ in the intermediate region. The dorsal pterygoid process and upper palatoquadrate cartilages are present in both single mutants. Ventrally in both, Meckel’s cartilage is extremely reduced. The intermediate region in WT forms the joint between the palatoquadrate and Meckel’s elements and a prominent retroarticular region of Meckel’s cartilage adjacent to the joint. In the *edn1* mutant both features are absent. In the *hand2* mutant the joint is absent, but in the territory of the retroarticular process we see a locally expanded wedge- or fan-shaped region of cartilage, previously interpreted as being dorsalized [10]. Not shown in this preparation, the bilateral fan-shaped regions often meet in the midline [10].

The zebrafish *edn1;hand2* double mutant has not been previously described. The most striking aspect is its overall phenotypic resemblance to the *edn1* single mutant. This finding is consistent with previous understanding that the two genes act positively with respect to ventral cartilage development, and that they function on the same genetic pathway [10], [12]. We do not see a substantially more severe phenotype that might result in the double mutant if the genes were on two different pathways and interacted synergistically. In the intermediate regions of both arches, where the single mutant phenotypes differ, the double mutant phenotype clearly most resembles the *edn1* mutant rather than the *hand2* mutant. In the second arch, the symplectic and joint are absent. Strikingly, in the intermediate region of the first arch where the single mutants perturb the WT condition in opposite directions (a reduction of cartilage in *edn1^−^* versus a local expansion in *hand2^−^*) we also see an *edn1^−^* -like reduction, showing that for this phenotype *edn1* is epistatic to *hand2*. This finding indicates that the relationship between the two genes is complex, seemingly in opposite directions for the positive interaction responsible for overall cartilage reduction and for the negative interaction in expansion in the first arch intermediate region (see Discussion).

### A Novel Interaction between *edn1* and *hand2* in the Embryonic Pharyngeal Arches

**Figure 2:**
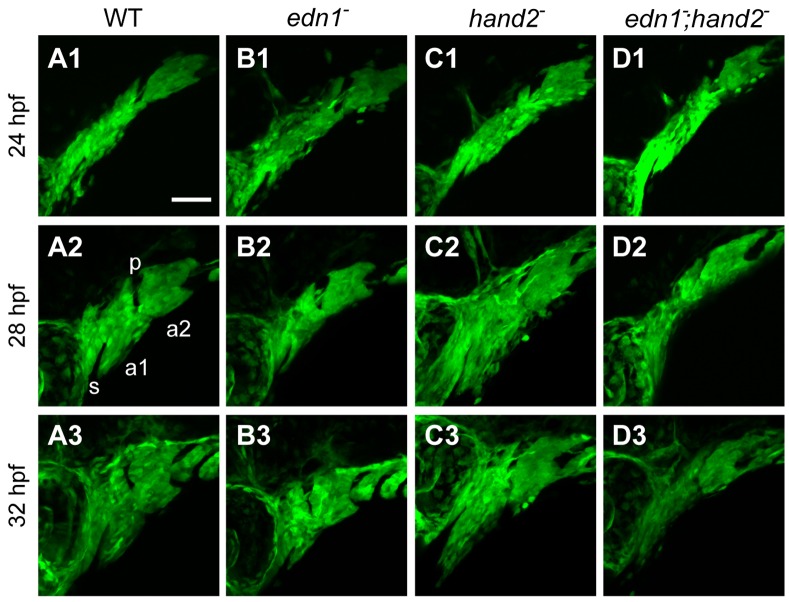
*edn1* and *hand2* are required for proper growth and morphogenesis of the early pharyngeal arches. Projections of lateral confocal images of embryos expressing *fli1a:EGFP* taken at 24, 28, and 32 hpf. This figure provides an overview of changes in the first two arches (a1, a2, separated by the first pharyngeal pouch, p) that are quantified and more fully documented in subsequent figures. Dorsal is to the top in each panel, and anterior is to the left. No differences in arch morphology are observed among the four genotypes at 24 hpf (upper row of panels). Subsequently the arches prominently lengthen along the DV axis; this DV extension is evidently reduced in the *edn1* mutant (B2,B3) and *edn1;hand2* double mutant (D2,D3) compared to WT and the *hand2* mutant. The arches (particularly the first), also shorten along the AP axis but there appears to be no differences in AP shortening among the genotypes. The *hand2* mutant also shows marked expansion of mesenchyme ventral to the stomodeum (s) in the anterior first arch (C2,C3). Scale bar: 50 µm.

**Figure 3:**
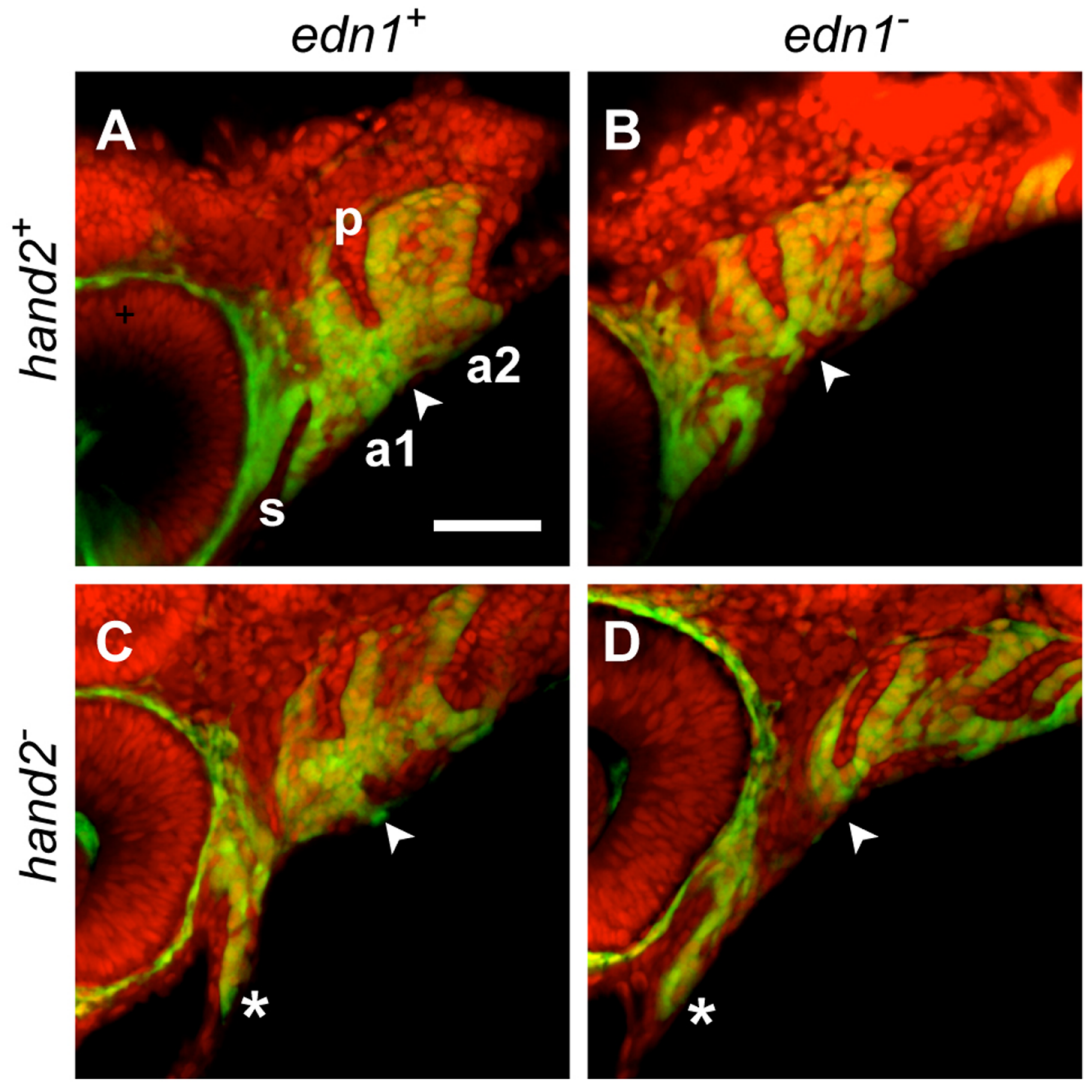
Counterstaining the *fli1a:EGFP*-expressing pharyngeal arches (a1, a2) with the nucleic acid stain SYTO 59 (red) clarifies the ectomesenchymal phenotypes (double-labeled). The DV ventral extension differences (arrowheads) are apparent ventral to the red-stained first pharyngeal pouch (p). Compared to the WT (A) this ventral region of mesenchyme is reduced in the *edn1* mutant (B) and double mutant (D), and expanded in the *hand2* mutant (C). Anterior extension of mesenchyme ventral to the stomodeum (s) is prominent in the *hand2* mutant and double mutant (*, C, D).

To explore the developmental basis of *edn1*– *hand2* interaction, we examined expression of *fli1a:EGFP*, a marker of neural crest derived ectomesenchyme, during early pharyngula stages (at 24, 28, and 32 hours postfertilization (hpf)). At these stages patterning genes under control of *edn1*, including *hand2*, are transcriptionally upregulated and likely functional [12]. Walker et al., using *dlx2a* expression as a proxy for arch morphology, discovered an *edn1* mutant arch phenotype that becomes apparent during this period – a ventral-specific reduction in the DV extent of the pharyngeal arches, which normally are rapidly extending along this axis [23]. At 24 hpf we find no differences in arch morphologies between the WT, the *edn1* and *hand2* single mutants, and the *edn1;hand2* double mutant (Figure 2, first row of panels). However, within the next four hours, prominent DV extension has occurred in the WT (compare Figure 2A1 and A2), and still more prominently in the *hand2* mutant (Figure 2C1 and C2). In contrast, there is reduced DV extension in the *edn1* single mutant (Figure 2B1 and B2, matching our earlier findings [23]). The 28 hpf DV extension in the *edn1;hand2* double mutant phenotype (Figure D2) is similar to that of the *edn1* single mutant, and note that the reduction in these mutants is opposite to the expansion in the *hand2* single mutant (Figure 2C2). These relations persist at the 32 hpf stage (third column of panels in Figure 2; Figure 3).

**Figure 4:**
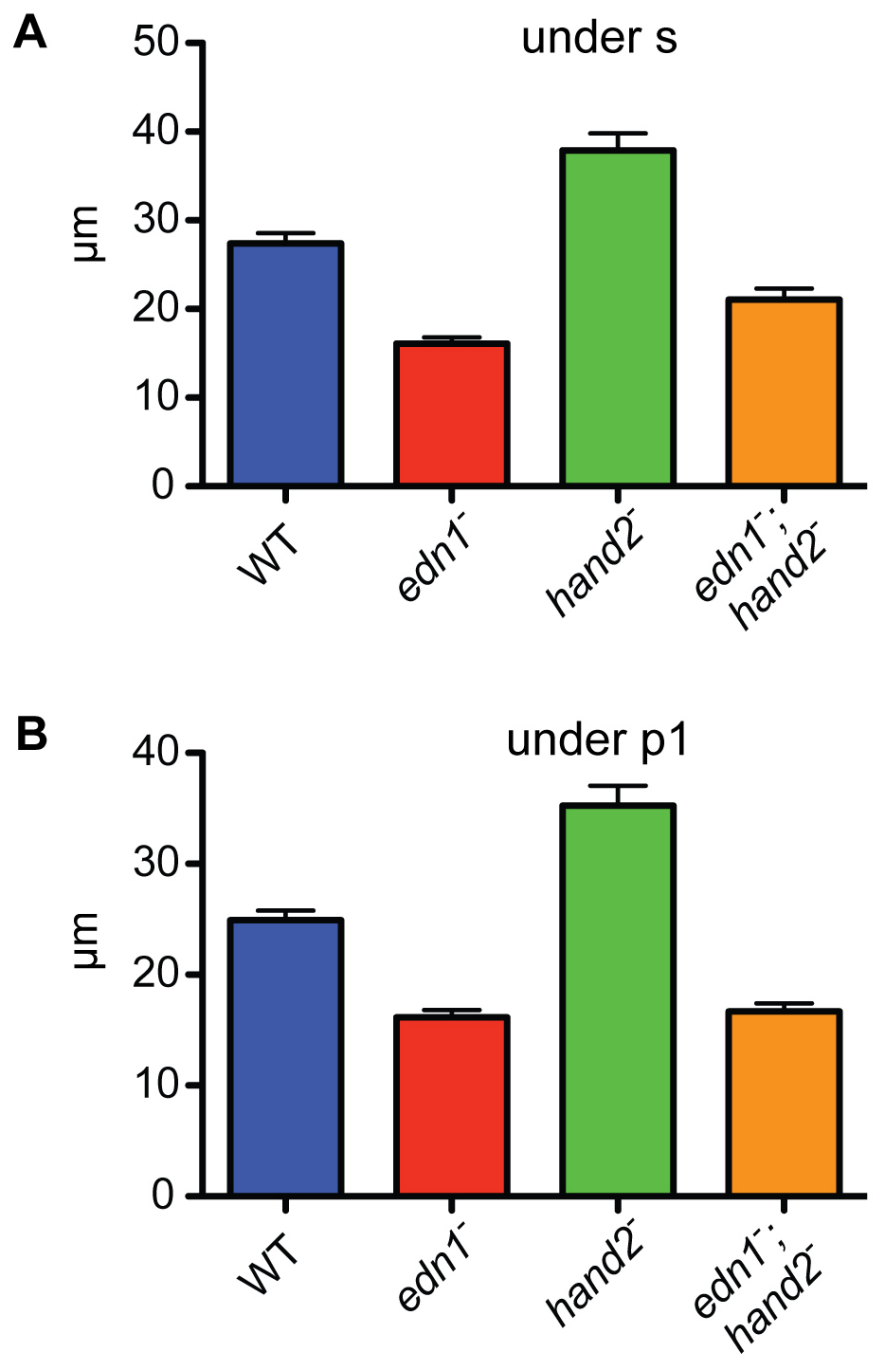
Quantification of the DV extension phenotypes supports *edn1* being epistatic to *hand2* in a single regulatory pathway. DV-length measurements were made for each group, beneath the end of the invaginated stomodeum (A) and beneath the first pharyngeal pouch (B, location indicated by arrowheads in Figure 3). In both locations, and compared to the WT, the *edn1* mutant shows reduced DV extension, the *hand2* mutant shows increased DV extension and the double mutant most closely resembles the *edn1* mutant. Tukey-Kramer analysis supports these three groupings as being significantly different (P<0.05), whereas the differences between *edn1^−^* and the double mutant are insignificant.

At two levels along the AP axis, we can learn whether the differences in DV extension of the arches are predominantly due to changes of the more ventral region of ectomesenchyme. One of the regions, examined previously [23], is the border region between the first and second arches, ventral to the first pharyngeal pouch (arrowheads in Figure 3). The second region is the anterior part of the first arch, where the invaginated oral ectoderm, the stomodeum, is ventrally underlain by the ectomesenchyme (Figure 3C, D; asterisk). In both of these regions epithelium forms a dorsal border to the mesenchyme, providing for quantification of the amounts of DV extension of these ventral mesenchymal regions. Such measurements in sets of 28 hpf genotyped embryos reveals that in both locations the *edn1* mutant phenotype has a significant reduction of the ventral mesenchyme compared with WT, the *hand2* mutant has an expansion, and the double mutant matches *edn1*, showing *edn1* to be the epistatic gene (Figure 4A, B). We note that these early phenotypes, as well as the epistatic relationship, are just the same as for the later first arch intermediate region skeletal phenotypes described above.

### The Pharyngeal arch DV Extension Phenotypes are likely due to Changes in arch Size

Two prominent possibilities for the differences in morphology we observed are that the cell arrangements in the pharyngeal arches differ according to genotype, hence giving rise to different arch shapes without a change in size, and/or that arch sizes are different, e.g., through changes in cell growth or death. We used several approaches to examine these possibilities.

**Figure 5:**
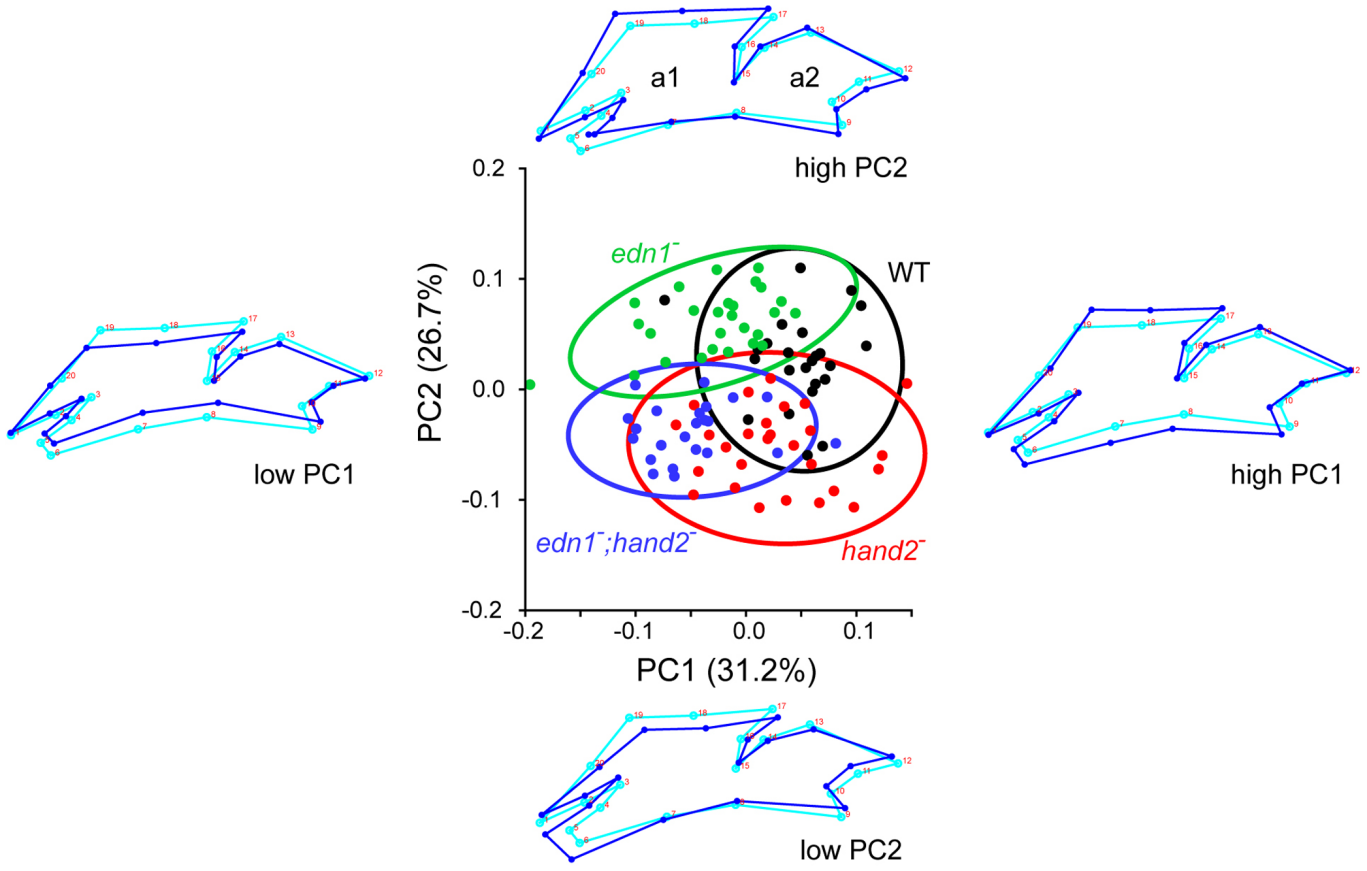
Quantification of arch morphology by PCA reveals that *edn1* and *hand2* regulate two prominent features of arch shape, DV extension (PC1) and ventral arch 1 anterior extension (PC2). Together these two deformations account for 58% of the total shape variation within the dataset, which uses landmarks (numbered in the accompanying wireframe diagrams) to outline the *fli1a:EGFP*-expressing tissue of the first two pharyngeal arches at 28 hpf. Shape change along the AP axis is minimal. We interpret the nature of the shape changes from the wireframes. For each, the light blue wireframe shows the consensus configuration and the dark blue wireframe shows the deformation associated with change (+ or -) along a PC axis. In the plot itself the individual measurements are grouped by genotype with a different color and 95% ellipses for each (sampling 23 or more individuals in each group). The data show overlap among the groups, yet Procrustes distance measurements reveal all the groups are significantly different from one another (P<0.0001 by permutation; data not shown). WT and the *hand2* single mutant score high on PC1, whereas the *edn1* single mutant and the double mutant score low on PC1. WT and the *edn1* mutant score high on PC2, whereas the *hand2* mutant and the double mutant score low. Hence the double mutant phenotype is additive in this analysis, combining the shape features of both single mutants.

Cell rearrangement could underlie convergence and extension of the arches during the early pharyngula period, evident from the time course shown in Figure 2. At 24 hpf the arches are not only very short along the dorsal-ventral axis, but they are also prominently elongated along the AP axis. By 28 hpf they have converged, i.e., shortened, along the AP axis and extended, or lengthened, along the DV axis. Do the arch shapes differ significantly according to genotype at this stage? We used the sensitive methods of landmark-based geometric morphometrics [25], [26], including a Principal Component Analysis (PCA) to compare the shapes of the arches. Plotting PC1 by PC2 yields a ‘shape space’ accounting for about 57% of the total shape variation (Figure 5). As shown by the shape configuration diagrams accompanying the plot, PC1 prominently captures the overall lengths along the DV axis of both arches, DV lengthening at positive PC1 values, and shorting at negative PC1 values. PC2, in contrast, primarily accounts for a more local change in the amount of tissue ventral to the stomodeum in the first arch (the region examined in Figure 4B). Notably, at this 28 hpf time point neither PC1 nor PC2 reveal substantial variation associated with arch lengths along the AP axis. If the basis of the shape change revealed by the PCA were due to AP convergence coupled with DV extension, we would expect an AP change to be detected by the PCA. Hence the findings suggest that the differences between genotypes with low PC1 values (*edn1* mutants and the double mutants), and high PC1 values (WT and *hand2* mutants) are not due to convergence and extension. The data do not show that arch convergence and extension is absent at this stage, only that the four genotypic groups do not differ from one another in this respect. Indeed, following the time course of changes by PCA suggests that the early arches are indeed undergoing convergence and extension, and that the genotype-specific changes occur progressively with time (Figure S1).

**Figure 6:**
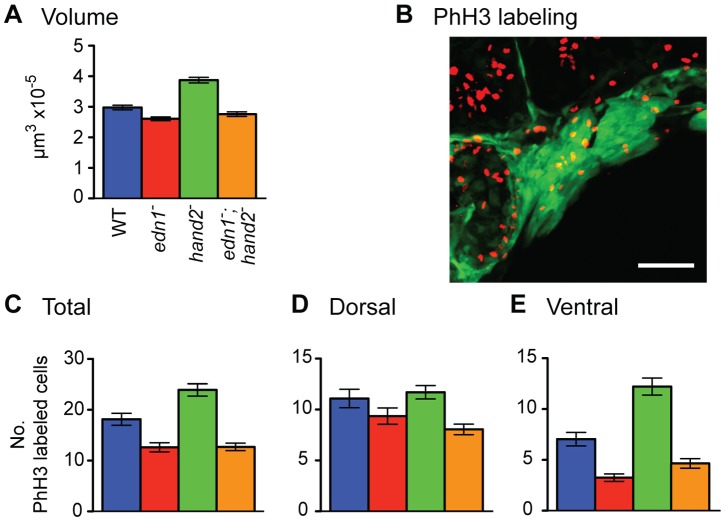
*edn1* positively and *hand2* negatively regulate the volume of pharyngeal arch *fli1a:EGFP* expressing mesenchyme (A) and ventral domain proliferation of neural crest derived cells (B–E). Data are for 28 hpf, a minimum of 23 individuals of each genotype were sampled. (B) shows an example 2-color image of phospho-histone H3 labeling for mitosing cells (red) and *fli1a:EGFP* expression (green). (C–E) show mean counts of double-labeled cells ±SEM. Tukey-Kramer comparison of the mean volumes (in A) reveals that the *edn1* mutant volume is significantly lower than WT, and that the hand2 mutant volume is significantly higher (P<0.05). Statistically the double mutant phenotype is neither different from the *edn1* mutant nor WT, suggesting some degree of phenotypic rescue of arch volume when *hand2* does not function. Tukey-Kramer analysis of the levels of proliferation in both the total mesenchyme (C) and ventral sector of mesenchyme (E) reveals three statistically distinctive classes, WT, the single *hand2* mutant, and the single *edn1* plus *edn1;hand2* mutant class (P<0.05). Genotypic differences for the dorsal mesenchyme (D) are insignificant.

Size variation is factored out of these geometric morphometric analyses (Figure 5), which examine only shape variation. Nevertheless, we interpret the PCA to mean that size change accounts for the changes in DV extension. We hypothesize that the genotype-specific differences revealed by PC1 specifically are simply accounted for by how much arch mesenchyme is present at this stage in the arches globally and/or in the ventral regions specifically. To test the hypothesis directly we measured the volumes of the arches, and predicted that arch volume should be greater in the *hand2* mutant relative to WT, less in the *edn1* mutant, and less than WT in the double mutant as well. No such changes in volume are predicted by the alternative, morphogenetic movement hypothesis. Quantitative estimates of the combined arch 1 and 2 volumes of regions of *fli1a:EGFP*-expressing mesenchyme, match these predictions (Figure 6A).

### *edn1* and *hand2* Interact to Control Cell Proliferation in the Ventral Pharyngeal Arches

The changes in the volumes of the neural crest-derived portions of the 28 hpf arches described above might arise through a variety of mechanisms, including differences in recruitment, cell death, cell size, or cell proliferation. We could not meaningfully test cell size because of the very tight packing of cells in the *fli1a:EGFP* expressing mesenchyme, however there is no indication of a prominent cell-size difference among these genotypes. TUNEL labeling showed that there is essentially negligible programmed cell death in the WT, and this level is not increased in the *edn1* mutant (Table S1), matching previous findings with *ednra1* and *ednra2* knockdown [27]. Additionally, we generated *edn1;p53* double mutants, and examined their early larval skeletal phenotypes (Figure S2). We found no phenotypic rescue. Hence, this analysis argues against p53-dependent cell death as an explanation for the reduced size of the arches.

We used phospho-histone H3 labeling to examine proliferation, including *fli1a:EGFP* as a second label to mark neural crest derived mesenchyme (Figure 6B). Double-labeled cells are present in both dorsal and ventral sectors of the arches (Figure 6B). Strikingly, the relative levels of proliferating ectomesenchymal cells within the total combined arch1 and 2 for each genotype are similar to those for arch volume (Figure 6C, compare with A). The differences between the single mutants are even more striking when proliferation in just the ventral part of the arches is compared, and the double mutant is statistically indistinguishable from the *edn1* single mutant (Figure 6E). Genotype-specific differences are less prominent in the dorsal sector (Figure 6D). These finding suggest that changes in the volume of ectomesenchyme in the pharyngeal arches that are associated with genotype are at least in part due to differences in cell proliferation, primarily occurring in the ventral regions. For all of these phenotypes, *edn1* and *hand2* exert their controls in opposite directions (positive versus negative) and the epistasis between the two genes is the same; *edn1* is epistatic to *hand2*.

### *hand2* Regulates Anterior Extension in the Ventral First arch in an *edn1*-independent Manner

**Figure 7:**
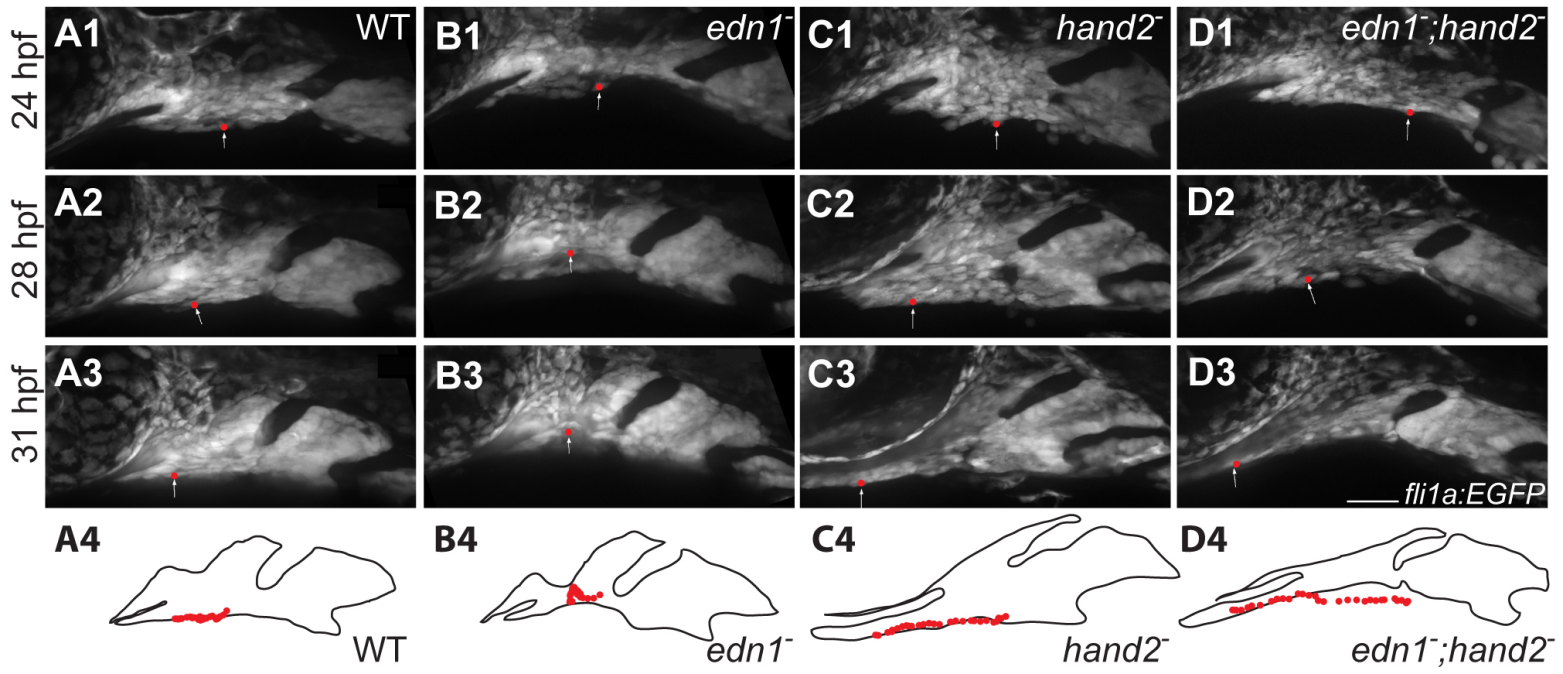
*hand2* negatively regulates anterior flow of ventral arch 1 cells. *fli1a:EGFP* animals were imaged by time lapse microscopy from 24–32 hpf. Panels are excerpts from the movie S1 in which maximum confocal projections are presented. Red balls represent the location of an individual cell that was manually tracked in each frame. The positions of tracked cells are overlaid in the outlines. Cells travel from near the midpoint of arch1 to a location posterior to the stomodeum and the eye in wild types and *edn1* mutants (A, B). Cells originating at a similar location in *hand2* and *hand2;edn1* double mutants travel much further, to a position well under the stomodeum and the eye (C, D). Scale bar: 50 µm.

Besides DV expansion of the ventral arches, as noted above we observed a prominent anterior extension of the ventral first arch shared by the *hand2* single mutant and the *edn1;hand2* double mutant (Figure 2; and the anterior extension is captured by PC2 in Figure 5 and Figure S1). This change is due to an expansion in the region ventral to the stomodeum, as might be due to a flow of cells into this ventral territory. We used time-lapse recordings to examine this hypothesis. We observed that in the WT and in all three mutant conditions there is a distinctive anterior flow of cells within the ventral first arch that serves to anteriorly extend the region singled out by PC2 (Figure 7, movie S1). This anterior extension movement is much more prominent in the *hand2* mutant and in the double mutant than in both WT and the *edn1*mutant. Following the courses of individual cells in the ventral arch (Figure 7, movie S1; arrows) strongly supports this inference. Our recordings suggest that WT and the *edn1* mutant form a single phenotypic class in which the anterior extension movement is relatively slow, whereas the *hand2* and double mutant share another class with much more rapid movement. We interpret these findings to mean that *hand2* functions as a negative regulator of this anterior extension cellular movement in the ventral first arch, and that *edn1* plays no role in this regulation.

## Discussion

Previous studies on the role of the Edn1 signaling pathway in development have mostly focused on the regulation of developmental patterning in the anterior pharyngeal arches: how are separate skeletal elements and their articulating joints specified in a position-dependent manner? Here we focused primarily on growth and morphogenesis in the embryonic pharyngeal arches. In doing so, we discovered a novel genetic interaction between *edn1* and *hand2.* This interaction shows itself in the control of very early DV outgrowth in the ventral arches during the pharyngula period of embryonic development. The control is mediated, at least in part, through *edn1*-dependent cell proliferation. However, we cannot rule out alternative mechanisms such as recruitment of additional cells to express *fli1a:EGFP*. Also novel is that we discovered *edn1* to be epistatic to *hand2* in a negative genetic interaction controlling this outgrowth and proliferation. In contrast, previous studies in zebrafish and mouse support both *edn1* and *hand2* as being positive, not negative, effectors of ventral arch outgrowth and differentiation. Finally, we show that *hand2* acts as a negative regulator of a previously undescribed morphogenetic movement that sweeps cells forward in an anterior direction within the ventral first pharyngeal arch. *edn1* appears to play no role in this ‘anterior extension’ cell movement, at least at the early stages of arch morphogenesis we examined.

### *hand2* as a Negative Regulator of *edn1*-dependent Cell Proliferation

We find that one *edn1* function is to promote early expansion of the ventral regions of the pharyngeal arches by upregulating local proliferation, and that this action is blocked by *hand2*. Consistent with previous zebrafish results with knockdowns of the *ednra* receptors [27], we find TUNEL labeling of cells in the zebrafish pharyngeal arches to be rare, and no change in either the *edn1* or *hand2* single mutant, suggesting that the cell death pathway is not involved in size regulation. These data contrast with some from other vertebrates suggesting *Edn1* and *Hand2* function positively together to regulate growth in arch size through inhibiting cell death [5], [7], [28], [29]. However, this mechanism remains controversial as work with *Hand2* conditional knockouts [13] suggests the cell death observed in *Hand2* conventional knockouts [5] may be due to loss of embryo viability, as opposed to a more direct function of *Hand2* in preventing cell death. Another possibility accounting for the difference between our data and those from other models is timing, for we focused on an early eight hour interval in the embryo when the mutant phenotypes are only first showing up. From the larval skeletal phenotypes it is likely that size regulation changes dynamically during arch development (discussed further below). We also note that in *in* *vitro* studies in human cancer cell lines EDN1 promotes cell proliferation as well as inhibiting cell death [30], [31], [32].

**Figure 8:**
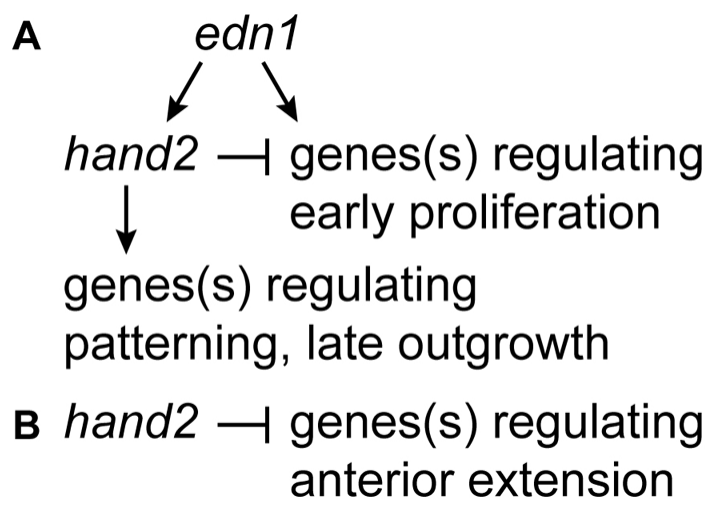
Regulatory genetic network for control of growth and morphogenesis in the pharyngeal arches by edn1 and hand2. (A) The model assumes that regulation of early proliferation in the ventral arches, in which *edn1* is epistatic to *hand2,* can account for the DV extension phenotype explored in this study. The positive pathway regulating patterning and late outgrowth accounts for the general reduction of elements in the ventral skeleton, as well skeletal disruption because of downregulation of downsteam patterning genes studied elsewhere [10], [12], [23], [24]. This model predicts a temporally dynamic switching in *hand2* function, from negative to positive, which can be explored in future studies. (B) We propose *hand2* negatively regulates the anterior extension movement in the ventral first arch independent of *edn1* function.

*edn1* being epistatic to *hand2* in the control of early proliferation would place *hand2 genetically* upstream of *edn1*, were their interaction within a linear genetic pathway [33]. In contrast, RNA in situ evidence is clear that *edn1* is *molecularly* upstream of *hand2* [6]. This apparent paradox can be easily accommodated by understanding that there is cross-talk among *edn1*-target genes [10], [12]. Hence genetic interaction is likely within a network, such as we propose in Figure 8A, rather than within a linear pathway. In fact, identical negative regulatory circuitry to that in Figure 8A has already been proposed: *Dlx* genes function in the intermediate domain under positive control of *edn1* as demonstrated by their downregulation in *edn1* mutants [6], [11], [12], [23], and the *Dlx* genes are negatively regulated by *hand2*, as demonstrated by their upregulation in *hand2* mutants [10], [13] The *edn1-hand2-Dlx* interaction thus provides precedent for our model in Figiure 8A, and indeed it is possible that Dlx genes themselves mediate the regulation of early proliferation that our model requires. Talbot et al. described the expansion of the ventral-anterior region of the first pharyngeal arch in the zebrafish *hand2* mutant being accompanied by an expansion of the intermediate domain *Dlx* genes [10]. Testing whether *hand2*– *Dlx* gene interaction is involved in ventral proliferation is likely possible with currently available methods, but would be difficult because of redundancy of function of *Dlx* genes (including *dlx3b, dlx4a* and *dlx5a* [10]), and lack of suitable loss of function mutants. Another class of homeobox genes, *Msx* genes, might also function as downstream effectors of *edn1*-dependent control cell proliferation; expression of at least two such genes are under positive control of *edn1* and negative control by *hand2* [12].

Our finding of negative interaction between *edn1* and *hand2* would at first glance be at odds with the previous understanding of positive interaction in zebrafish. The earlier model derives in part from the finding that the ventral elements of the early larval skeleton are generally reduced, hypoplastic, with loss of function of either of the two genes. In examining the early larva we also observe generalized ventral skeletal hypoplasia in all three mutant conditions we addressed, confirming the earlier work. Our model incorporates the positive interaction simply by adding a branch to the network (the downward branch in Figure 8A). The key difference between the two output branches of the network is likely one of timing, i.e., *hand2* regulation switches from negative to positive at stages later than the 24–32 hpf stages we examined here. In support, aspects of *hand2* function have already been shown to be dynamic during embryonic pharyngeal arch development [15], [23], [24].

### How does Control of Early Pharyngeal arch Phenotype Relate to the Later Cartilage Phenotype?

We observed that even though Meckel’s cartilage is severely reduced in *hand2* mutant larvae, matching previous studies [10], [12], there is a local size *increase* in this cartilage’s retroarticular process [10], [24]. The retroarticular region is that part of the cartilage nearest to the joint, i.e., the most dorsal or proximal region of this element. The retroarticular is not distinguished in the overall massively reduced Meckel’s cartilage of the *edn1* mutant, and the *edn1;hand2* double mutant matches the *edn1* mutant in this respect.

We propose that developmental regulation of the size of this most proximal region of the ventral cartilage occurs at the very earliest stages of pharyngeal arch development after neural crest migration. This is the time period when we have shown that negative control by *hand2* is exerted. This hypothesis predicts that the most proximal region of Meckel’s cartilage, a region within the intermediate domain of patterning [10] might be the developmentally oldest region of the cartilage. We do not know if the ventral cartilages grow out in some temporally-specific proximal-to-distal sequence, as could be examined in future studies.

### *hand2* Negatively Regulates *edn1*-independent Morphogenetic Movement

In addition to the DV outgrowth regulation just discussed, we observed a negative regulation by *hand2* of an anterior extension in the ventral first arch, most likely independent of *edn1* function (Figure 8B). Whereas our evidence strongly points to DV outgrowth being due to cell proliferation, morphogenetic movement is just as clearly the primary cause of anterior extension; our time lapse recordings show movement of ventral mesenchymal cells across the arch, flowing into the region of the stomodeum, and underneath the stomodeum. The recordings, along with our PCA, suggest this movement is specific to the ventral first arch; finding that cells move within the arch itself suggests active cellular migration. While it is possible that subtle changes in this morphogenesis are present in *edn1* mutants, we are unable to detect any *edn1* dependent differences in our time lapse analyses. Interestingly, some *Hand2* expression domains are apparently independent of Edn1 signaling in both zebrafish and mice [6], [8], and we propose that the small amount of *hand2* expressing cells that remain in *edn1* mutants are competent to prevent the morphogenetic movement. In fact, ectopic BMP can induce *hand2* expression in the absence of *edn1* [15], a precedent for *hand2* functioning *independent* of *edn1* in ventral arch1 development.

The time-lapse recordings also show a prominent anterior movement of the second arch, but not its narrowing, and seemingly not cells moving within the arch as for the first. Hence we interpret the second arch movement as being passively imposed by the first, to which it is connected through ventral mesenchyme. That cell migration is specific to the first arch makes sense functionally because of the way the invaginated oral ectoderm forms a barrier preventing ventrally migrating neural crest from populating the ventral anterior region of the arch directly: The anterior movement is required for the development of the lower jaw. No such epithelial barrier exists in the second or more posterior arches. We note that micrognathia is a relatively common human craniofacial disorder, a part of many syndromes (e.g, DiGeorge [OMIM188400], Treacher-Collins [OMIM154500],) and not well understood. It could well be that some specific instances of the human disorder could involve partial failure of anterior extension.

Our work not only identifies Edn1 as a growth factor in pharyngeal arch development, but particularly highlights the complexity of regulation involving *hand2*, both in terms of signaling pathways that converge on *hand2* and in its role as a focal point in the regulation of downstream effector genes mediating a variety of functions – patterning [10], [15], as well as growth and morphogenesis (Figure 8). In particular, our study demonstrates the value of the zebrafish model in allowing precise investigation of the cellular events occurring during early pharyngeal arch development, and how specific cellular behaviors depend on the developmental regulatory gene environment.

## Materials and Methods

### Fish Stocks and Maintenance

Fish were raised under standard conditions [34] and staged as described [35]. The following lines have been previously described: *edn1^tf216b^* [6], *Df(Chr01:hand2)s6* [36], Tg(*fli1a:EGFP)* [37], *p53^zdf1^* [38]. Heterozygous lines were maintained on an inbred AB genetic background. *edn1^−^* was identified using forward primer: 5′ GGTGCTCCAGCATCTTTGGGTC3′ and reverse primer: 5′TGTCTGTTCTGACTTACTCTGGTG3′ resulting in a 153 bp product. Digestion with MseI cleaved the PCR product from the mutant allele into 84 and 69 bp fragments. *hand2^−^* was identified by PCR using forward primer: 5′ GCGGACAGTGAAACGTAGACC 3′ and reverse primer: 5′ GCCTTTCTTCTTTGGCGTCTGTC 3′ resulting in a 257 bp product from the WT allele. *p53^−^* was genotyped as described [38] All of our work with zebrafish has been approved by the University of Oregon Institutional Animal Care and Use Committee (IACUC). Assurance number for animal research: A-3009-01.

### Skeletal Staining and Morphometric Analysis

Larvae were fixed at 4 or 6 days postfertilization (dpf) and stained with Alcian Blue for cartilage and Alizarin Red for bone as described [39], [40].

### Microscopy

Skeletal preparations were imaged on a Zeiss Axiophot 2. Static confocal images were captured on a Zeiss LSM 5 Pascal as previously described [41]. Briefly, embryos were manually dechorionated, anesthetized with tricaine and fixed in 4% paraformaldehyde in PBS overnight at 4°C. A piece of the tail was removed and used for genotyping each sample. Embryos were washed with PBS and transferred to 6% methylcellulose on a microscope slide. Z-stacks totaling approximately 90 µm were imaged at 1.5–2.5 µm intervals. For time-lapse movies, animals were mounted and imaged as described [24], [42] on a Leica SD6000 spinning disk confocal. Movies were assembled using Metamorph (Molecular Devices) and ImageJ.

### Geometric Morphometric and Volume Analyses

Confocal images taken at 24, 28, and 32 hpf were corrected for orientation using 3D rendering in Volocity (PerkinElmer). For geometic morphometric analysis, twenty landmarks outlining the *fli1a:EGFP* expressing ectomesenchyme of the first two pharyngeal arches (PAs) were digitized at the positions shown in Figure 5 using tps Dig, version 2.04 software [43]. Further analyses were done in MorphoJ [25], [26]. The data were Procrustes aligned to remove size, rotation, and translation effects, and the shapes analyzed by PCA and Discriminant Function Analysis. Combined volumes of these two arches were measured using Volocity. Regions of interest were established around the anterior PAs and volume measurements were of regions expressing *fli1a:EGFP*. To provide a counterstain showing total cells within the arches (as in Figure 3), the embryos were incubated with the nucleic acid stain SYTO 59 (Invitrogen) at a 1∶1000 dilution in PBSTx (1% Triton X-100 in 1x PBS) for 1 hour at room temperature. The embryos were washed for 15 minutes with PBSTx at room temperature.

### Cell Proliferation Assay

Proliferating cells were detected using anti-phospho-histone H3 antibody. Embryos were manually dechorionated, anesthetized with tricaine, and fixed in 4% paraformaldehyde in PBS overnight at 4°C. Prior to staining, embryos were washed with PBSTx and blocked in 10% normal goat serum (NGS) in PBDTx (0.1% Triton X-100, 1% DMSO, 1% BSA in 1x PBS) for at least 2 hours. Embryos were incubated with rabbit anti-phospho-histone H3 (Millipore) at 1∶1000 dilution in blocking solution overnight at 4°C, washed with PBSTx, then incubated with Alexafluor 546 goat anti-rabbit IgG (Invitrogen) at 1∶500 dilution in PBSTx overnight at 4°C. Samples were visualized by confocal microscopy. Phospho-histone H3 positive cells were detected with Volocity. Regions of interest were set around the PAs and only cells positive for both phospho-histone H3 and *fli1a:EGFP* were counted.

### Cell Death Assay

TUNEL assay was performed to detect cell death. Embryos were dehydrated in 100% MeOH for 1 hour at -20°C then rehydrated by sequential incubation with 75%, 50%, and 25% MeOH in PBSTx at −20°C and with PBSTx at RT for 10 minutes each. Embryos were permeabilized by incubation in Proteinase-K (1 µg/mL) then fixed in 4% PFA for 20 minutes. Embryos were further permeabilized in Permeabilization Solution (0.1% Triton X-100, 0.1% sodium citrate) for 30 minutes then washed with PBSTx. DNA fragmentation was detected by incubation with terminal deoxynucleotidyl transferase and TMR dUTP for 1 hour at 37°C (Roche). Embryos were washed in PBSTx then imaged.

## Supporting Information

Figure S1 **The distinctive pharyngeal arch shapes of WT, edn1 and hand2 mutants arise progressively from 24 hpf to 32 hpf.** PCA as in Figure 5 of the main text, but here aligned separately for this developmental age series. Nevertheless this PCA and that shown in Figure 5 capture largely the same shape changes, as revealed by comparing the wireframes in each figure. A: PC2 by PC1 scatter plot with all of the individual samples plotted (a minimum of 20 in each of the nine groups). Gray-black filled circles represent WT, pink-red rectangles represent *edn1* mutants, and light to dark blue triangles represent *hand2* mutants. B. The same plot but showing the means for each genotype-age group. At the 24 hpf time point the three genotypes completely overlap. DV extension, captured by PC1, then increases markedly with developmental age for all genotypes, with *edn1^−^* lagging behind WT and *hand2^−^*. The *hand2* mutant shows progressive expansion in the ventral-anterior arch 1 region captured by negative PC2, whereas WT and *edn1^−^* show slight change in the opposite direction.
(TIF)

Figure S2 **Loss of function of the programmed cell death gene** ***p53*** **does not rescue the skeletal phenotype of the** ***edn1*** **mutant.** Flat-mount of cartilage and bone stained with Alcian Blue and Alizarin Red. The skeletal phenotypes of the *edn1* single mutant and the *edn1;p53* double mutant appear identical, whereas phenotypic rescue would be expected if programmed cell death of pharyngeal arch precursor cells accounted for the *edn1^−^* hypoplastic skeleton. Hence the experiment argues against cell death as an explanation for the reduced size of the arches.
(TIF)

Table S1 **TUNEL labeling to detect cell death in the pharyngeal arches.**
(DOCX)

Movie S1 ***hand2*** **negatively regulates anterior flow of ventral arch 1 cells.** *fli1a:EGFP* animals were imaged by time lapse microscopy from 24–32 hpf. Movies are confocal projections in which individual cells were manually tracked at each frame (arrow). Cells travel from near the midpoint of arch1 to a location posterior to the stomodeum and the eye in wild types and *edn1* mutants (A, B). Cells originating at a similar location in *hand2* and *hand2;edn1* double mutants travel much further, to a position well under the stomodeum and the eye (C, D). Scale bar: 50 µm
(MOV)
