# Supplementary figures and images for: edn1 and hand2 Interact in Early Regulation of Pharyngeal Arch Outgrowth during Zebrafish Development
Source: PLoS One. 2013 Jun 24;8(6):e67522. doi: 10.1371/journal.pone.0067522 (PMC3691169; doi:10.1371/journal.pone.0067522)

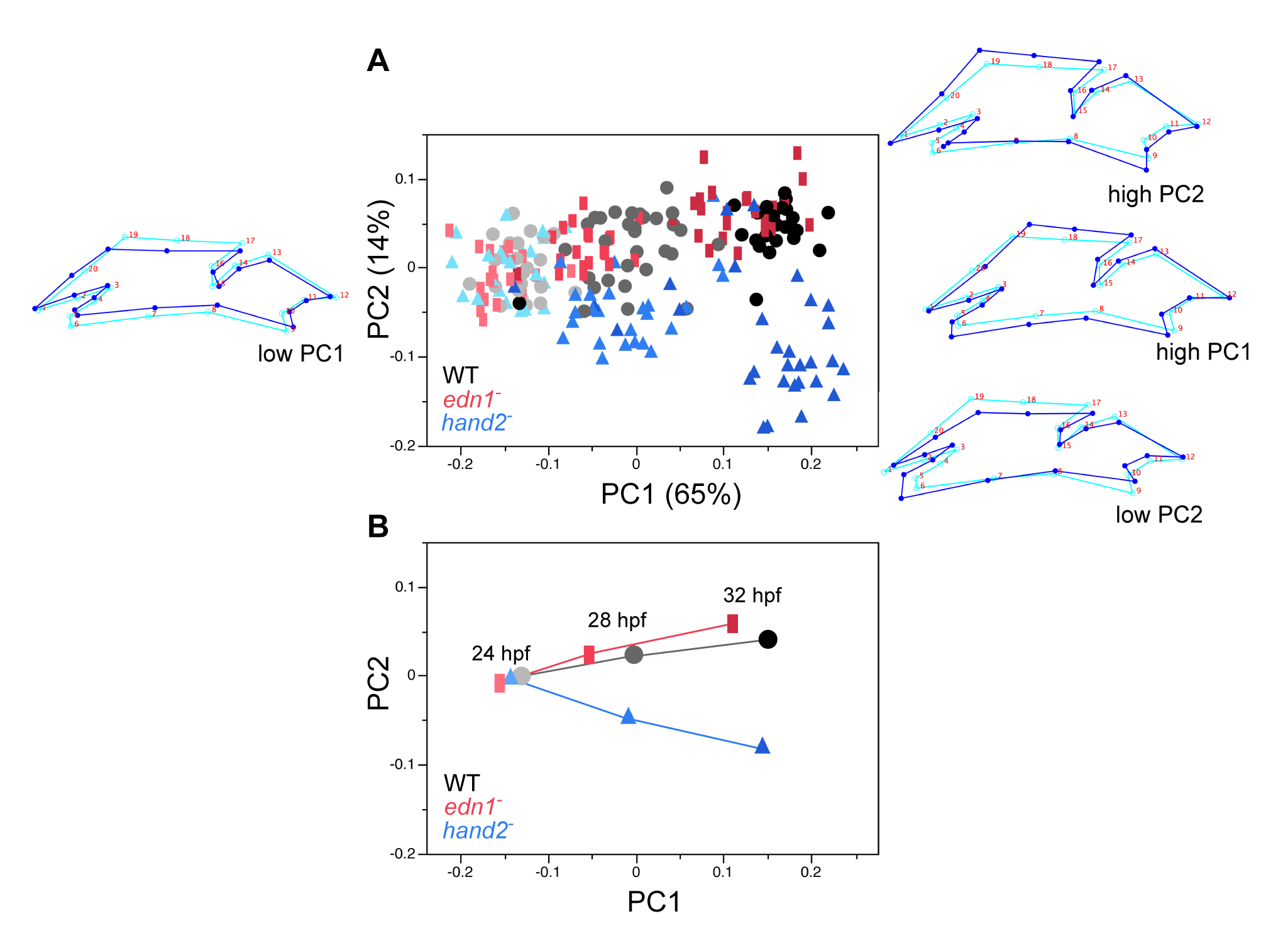

Supplement: Figure S1 — The distinctive pharyngeal arch shapes of WT, edn1 and hand2 mutants arise progressively from 24 hpf to 32 hpf. PCA as in Figure 5 of the main text, but here aligned separately for this developmental age series. Nevertheless this PCA and that shown in Figure 5 capture largely the same shape changes, as revealed by comparing the wireframes in each figure. A: PC2 by PC1 scatter plot with all of the individual samples plotted (a minimum of 20 in each of the nine groups). Gray-black filled circles represent WT, pink-red rectangles represent edn1 mutants, and light to dark blue triangles represent hand2 mutants. B. The same plot but showing the means for each genotype-age group. At the 24 hpf time point the three genotypes completely overlap. DV extension, captured by PC1, then increases markedly with developmental age for all genotypes, with edn1− lagging behind WT and hand2−. The hand2 mutant shows progressive expansion in the ventral-anterior arch 1 region captured by negative PC2, whereas WT and edn1− show slight change in the opposite direction. (TIF) [file pone.0067522.s001.tif]

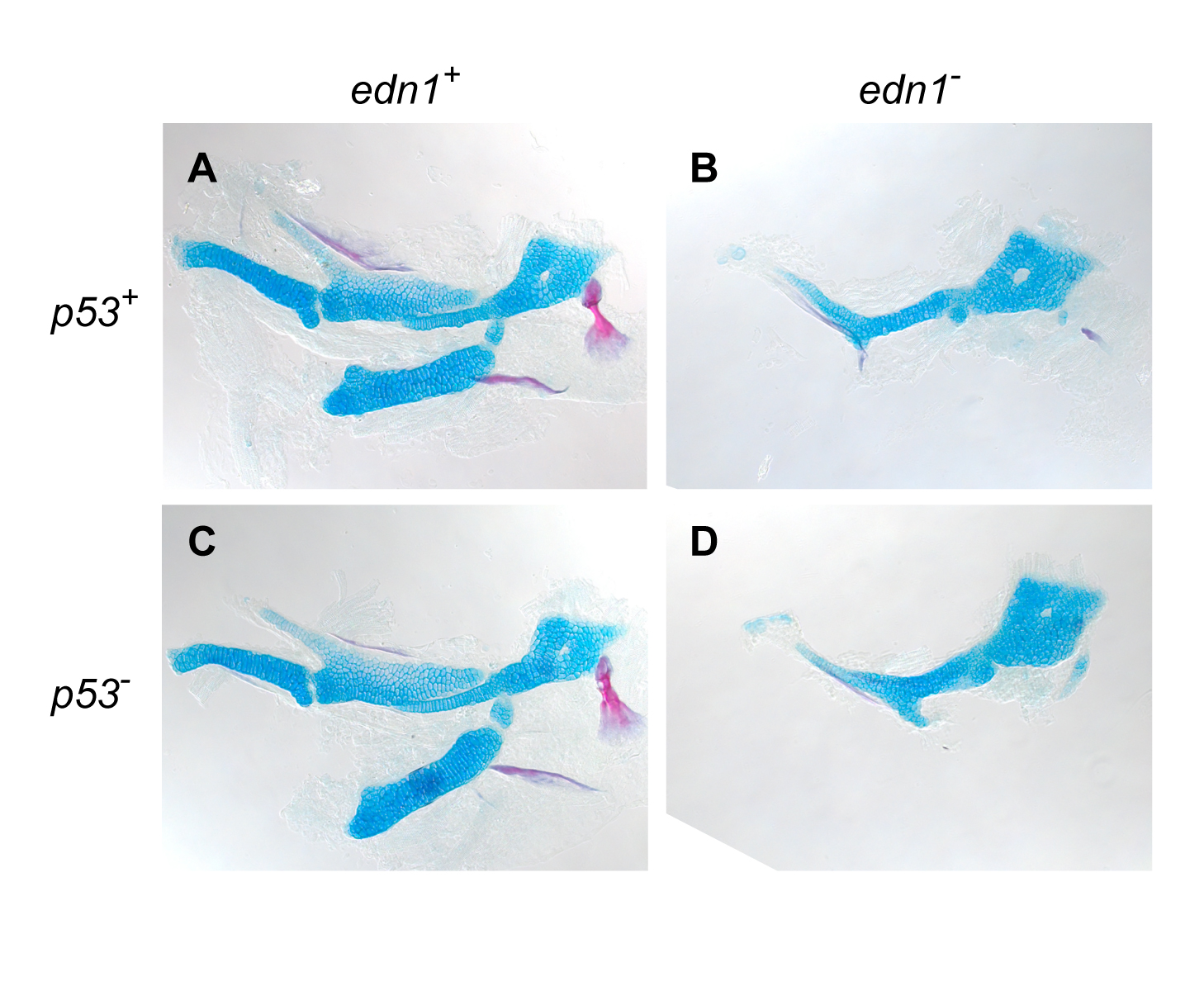

Supplement: Figure S2 — Loss of function of the programmed cell death gene p53 does not rescue the skeletal phenotype of the edn1 mutant. Flat-mount of cartilage and bone stained with Alcian Blue and Alizarin Red. The skeletal phenotypes of the edn1 single mutant and the edn1;p53 double mutant appear identical, whereas phenotypic rescue would be expected if programmed cell death of pharyngeal arch precursor cells accounted for the edn1− hypoplastic skeleton. Hence the experiment argues against cell death as an explanation for the reduced size of the arches. (TIF) [file pone.0067522.s002.tif]
