# Supplementary material for: edn1 and hand2 Interact in Early Regulation of Pharyngeal Arch Outgrowth during Zebrafish Development
Source: PLoS One. 2013 Jun 24;8(6):e67522. doi: 10.1371/journal.pone.0067522 (PMC3691169; doi:10.1371/journal.pone.0067522)
Supplement: Table S1 — TUNEL labeling to detect cell death in the pharyngeal arches. (DOCX) [file pone.0067522.s003.docx]

**Supplementary Table 1. TUNEL labeling to detect cell death in the pharyngeal arches.**

| hpf | WT  mean+SE (n)* | *end1^-^* | *hand2^-^* |
| --- | --- | --- | --- |
| 24 | 0.50+0.23 (12) | 0+0 (12) | 0+0 (13) |
| 28 | 0+0 (11) | 0+0 (11) | 0+0 (12) |
| 32 | 0.21+0.11 (14) | 0.36+0.15 (11) | 0.20+0.13 (12) |

*Quantification using Volocity, showing the total mean number of labeled cells (+ SE) in combined arch 1 and 2 ectomesenchyme at the time period shown. Only extremely few TUNEL positive neural crest derived cells were detected in the pharyngeal arches over the eight-hour time period.
